# Clinical and laboratory features of anti-MAG neuropathy without monoclonal gammopathy

**DOI:** 10.1038/s41598-019-42545-8

**Published:** 2019-04-16

**Authors:** Elba Pascual-Goñi, Lorena Martín-Aguilar, Cinta Lleixà, Laura Martínez-Martínez, Manuel J. Simón-Talero, Jordi Díaz-Manera, Elena Cortés-Vicente, Ricard Rojas-García, Esther Moga, Cándido Juárez, Isabel Illa, Luis Querol

**Affiliations:** 1 Neuromuscular Diseases Unit, Department of Neurology, Hospital de la Santa Creu i Sant Pau, Universitat Autònoma de Barcelona, Barcelona, Spain; 2 Centro para la Investigación Biomédica en Red en Enfermedades Raras (CIBERER), Madrid, Spain; 3 Department of Neurology, Hospital de la Santa Creu i Sant Pau, Universitat Autònoma de Barcelona, Barcelona, Spain; 4 Department of Immunology, Hospital de la Santa Creu i Sant Pau, Universitat Autònoma de Barcelona, Barcelona, Spain

## Abstract

Antibodies against myelin-associated glycoprotein (MAG) almost invariably appear in the context of an IgM monoclonal gammopathy associated neuropathy. Very few cases of anti-MAG neuropathy lacking IgM-monoclonal gammopathy have been reported. We investigated the presence of anti-MAG antibodies in 69 patients fulfilling diagnostic criteria for CIDP. Anti-MAG antibodies were tested by ELISA and confirmed by immunohistochemistry. We identified four (5.8%) anti-MAG positive patients without detectable IgM-monoclonal gammopathy. In two of them, IgM-monoclonal gammopathy was detected at 3 and 4-year follow-up coinciding with an increase in anti-MAG antibodies titers. In conclusion, anti-MAG antibody testing should be considered in chronic demyelinating neuropathies, even if IgM-monoclonal gammopathy is not detectable.

## Introduction

Polyneuropathy associated with IgM monoclonal gammopathy of uncertain significance (MGUSP) is a rare form of chronic immune-mediated neuropathy. More than 50% of these patients harbor antibodies against myelin-associated glycoprotein (MAG)^1,2^. Patients with anti-MAG+ MGUSP present with a predominantly sensory neuropathy with ataxia and tremor with poor response to immunotherapy^3^.

Anti-MAG antibodies were described to be invariably associated with IgM monoclonal gammopathy^4^, and clinical practice guidelines recommend to test them in patients with detectable IgM monoclonal gammopathy^5^. Anecdotal cases of neuropathy with anti-MAG antibodies lacking monoclonal gammopathy were reported^6–8^. A recent Japanese study^8^ reported a prevalence of 5.6% of anti-MAG positive patients in a cohort of 36 patients with chronic demyelinating polyneuropathy with no monoclonal gammopathy. Antibodies in these patients were tested by enzyme-linked immunosorbent assay (ELISA) and confirmed by Western blot analysis.

Here we investigate the presence of anti-MAG antibodies in patients fulfilling diagnostic criteria for chronic inflammatory demyelinating polyradiculoneuropathy (CIDP) without IgM monoclonal gammopathy. Also, we describe the clinical, electrophysiological and laboratory findings of four patients with anti-MAG associated neuropathy without any detectable monoclonal gammopathy at the time of diagnosis.

## Results

### Patients

**Figure 1:**
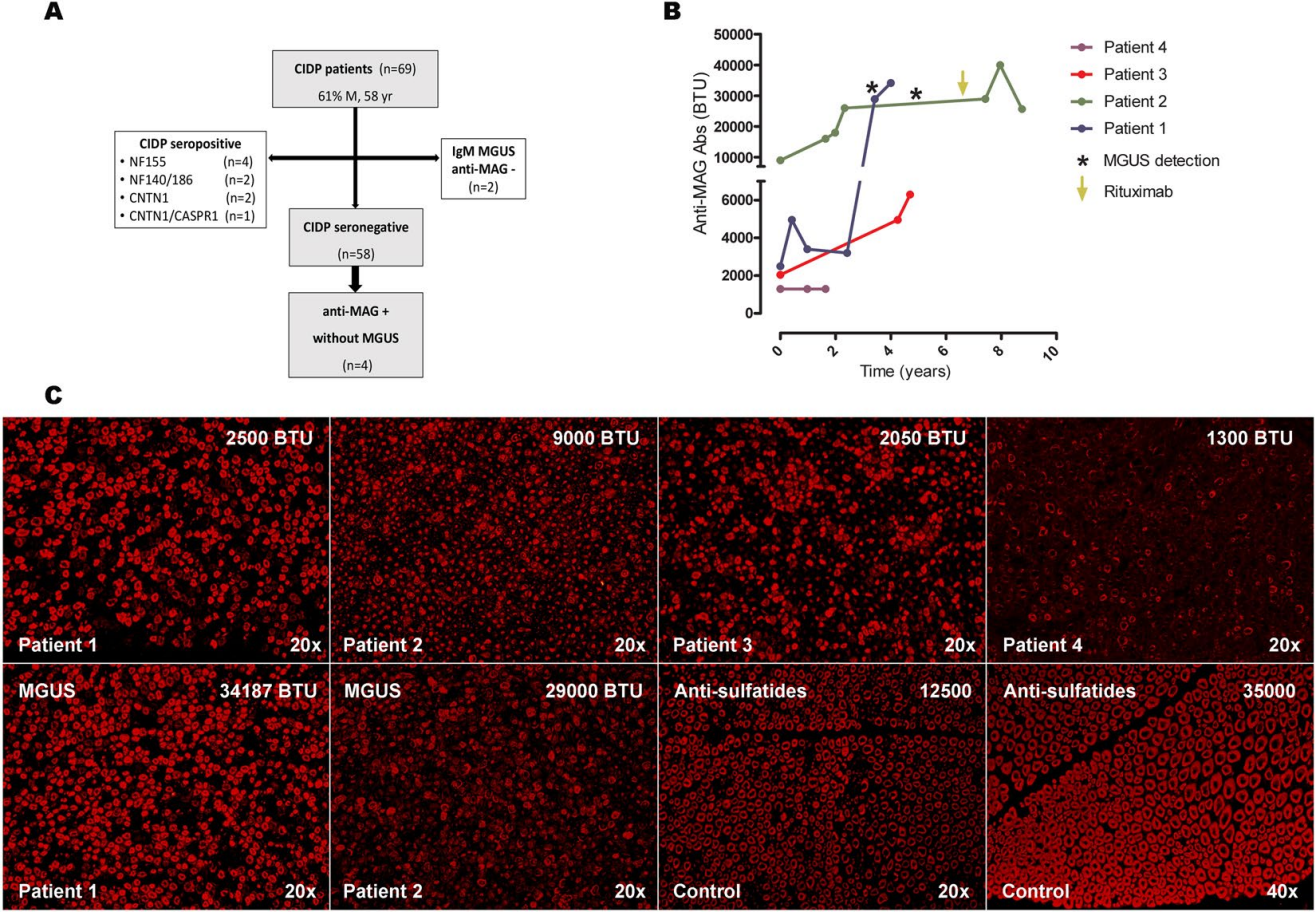
Flowchart of the study population (**A**). Serial anti-MAG antibody titers during follow-up (**B**). The asterisks highlight the detection of IgM MGUS in patients 1 and patient 2. The arrow indicates rituximab administration. Immunohistochemistry studies with serum from patients 1–4 showing IgM binding on the myelin sheaths. Immunofluorescence intensity increased in patients 1 and 2 after MGUS detection (**C**). Staining pattern of patients anti-MAG- sulfatides+ MGUSP used as control are shown. Titers of anti-MAG and anti-sulfatides antibodies are represented. (Anti-IgM, 20x and 40x original magnification). BTU Bühlmann test units; IgM immunoglobulin M; MAG myelin-associated glycoprotein; MGUS monoclonal gammopathy of uncertain significance.

We detected 69 patients (61% males, mean age 58 years) fulfilling CIDP diagnostic criteria. Flowchart of the study population is represented in Fig. 1A. Briefly, nine patients with antibodies toward NF155 (n = 4; 5.8%), NF140/186 (n = 2; 2.9%), CNTN1 (n = 2, 2.9%) or CNTN1/CASPR1 (n = 1; 1.4%), all of them negative for anti-MAG antibodies, were excluded from the seronegative cohort. Thirteen patients had monoclonal gammopathy (IgA n = 1; IgG n = 9; IgM n = 2; IgA + IgG n = 1) at diagnosis. The two CIDP patients with IgM monoclonal gammopathy were anti-MAG negative. Finally, we tested anti-MAG antibodies by ELISA in 58 CIDP seronegative patients. Anti-MAG antibodies were detected in four patients (6.9% of the seronegative patients; 5.8% of the whole CIDP cohort) without IgM monoclonal gammopathy.

### Clinical and neurophysiological features

Clinical and epidemiological features of all four patients are summarized in Table 1. All of them were males, with ages ranging from 58 to 70 years. Patients 1 and 2 presented with progressive distal sensory disturbances, while patient 4 presented with gait imbalance due to sensory ataxia. Patient 3 was diagnosed of essential tremor and had an incipient neuropathy with impaired vibration sensation in the lower limbs. Physical examination revealed mild to moderate sensory ataxia and mild to severe action tremor in all patients. During follow-up, patients 1, 2 and 4 developed distal motor involvement. Nerve conduction studies (Table 1) demonstrated prolonged distal motor latencies in patients 1 and 4 and mild to moderate reduction of motor or sensory nerve conduction velocities in all four patients. Also, F-waves showed prolonged latencies in patients 1, 2 and 3; and were absent in patient 4. Temporal dispersion was observed in patient 1, and compound muscle action potentials or sensory nerve action potentials were reduced in all four patients. An additional file shows nerve conduction studies in more detail (Supplementary Table 1).

**Table 1:** Summary of clinical and laboratory findings of patients with anti-MAG neuropathy without monoclonal gammopathy.

	Patient 1	Patient 2	Patient 3	Patient 4
Age at onset, yr; Sex	58; M	70; M	70; M	68; M
Past medical history	No	Hypertension, diabetes	Osteoarthritis, Essential tremor	Arthritis (methotrexate)
**Clinical manifestations**				
Initial symptoms	Distal sensory disturbance upper > lower limbs	Distal sensory disturbance upper > lower limbs	Postural tremor	Gait ataxia
Limb weakness	Distal > proximal mild	Distal mild	No	Distal > proximal moderate distal atrophy
Gait ataxia	Mild	Moderate	Mild	Moderate
Intention tremor	Mild	Moderate, upper limbs	Severe, head and upper limbs	Moderate, upper limbs
**Electrophysiological findings**				
Prolonged motor distal latencies	+	−	−	+
Reduction of NCV	+	+	+	+
Prolonged F-wave latencies	+	+	+	+
Conduction block	−	−	−	NA
Temporal dispersion	+	−	−	+
Reduced CMAPs	+	+	+	
Reduced SNAPs	+	+	+	+
**Laboratory findings**				
Cerebrospinal fluid findings (protein; cell count)	1,1 g/L; 2cells/mm^3^	1,4 g/L; 2cells/mm^3^	NA	0,44 g/L; 0cells/mm^3^
IgM levels * (presentation)	163 mg/dL	174 mg/dL	301 mg/dL	63 mg/dL
Anti-sulfatides and gangliosides	Negative	Negative	Negative	Negative
Anti-MAG Abs titers (presentation)	2500	9000	2050	1300
Monoclonal protein, levels (follow-up)	IgM-κ, < 1 g/L	IgM-κ, < 1 g/L	No	No
Malignancy screening	Negative	Negative	NA	NA
Treatment and response	IVIg: good	IVIg: partial azathioprine: no rituximab: good	IVIg: partial	IVIg: good steroids: no cyclosporine: no

*IgM normal values: 40–230 mg/dL.

CMAPs: compound muscle action potential; IVIg: intravenous immunoglobulin; κ: kappa light chain; M: male; NA: not available; NCV: nerve conduction velocities; SNAPs: sensory nerve action potential; yr: years.

All patients were treated with intravenous immunoglobulin (IVIg) (2 g/Kg), and good response was observed in patient 1 and 4, while partial response was observed in patients 2 and 3. Patient 2 was treated with azathioprine without response. Upon IgM MGUS detection, rituximab (375 mg/m^2^, once weekly for 4 weeks followed by 1 additional dose 1 month later) was started and we observed disease stabilization. IVIg were suspended in patient 4, due to toxicodermia and neither prednisone (1 mg/Kg/d), nor cyclosporine (125 mg/12 h) showed any significant benefit.

### Antibody assays

Anti-MAG antibodies tested positive at diagnosis in four patients by ELISA. Immunofixation did not detect monoclonal gammopathy at diagnosis in any of these patients, and total IgM levels were only mildly elevated in patient 3 (301 mg/dL, upper limit 230 mg/dL). Anti-MAG antibody titers and presence of IgM monoclonal gammopathy by immunofixation was tested periodically in these patients depending on their visit schedules. Follow-up anti-MAG antibody titers are shown in Fig. 1B. Antibodies to sulfatides and gangliosides were negative in all four patients.

In patients 1 and 2 we detected an IgM monoclonal gammopathy after 3 and 4 years of follow-up respectively **(**Fig. 1B**)**, while in patients 3 and 4 no monoclonal gammopathy has been detected yet (follow-up of 5 and 2 years respectively). In patient 1, the detection of monoclonal gammopathy coincided with a significant increase in anti-MAG antibody titers. At that time, hematological malignancy screening tests performed in patients 1 and 2 were negative. Both had a serum IgM-kappa monoclonal protein of less than 1 g/L, a negative Bence-Jones protein urine test, and a radiographic X-ray skeletal survey without bone lesions. Accordingly, both patients were diagnosed of IgM MGUS and underwent hematological follow-up. Neither of them developed malignancy to date.

### Immunohistochemistry

At diagnosis, serum from all four patients showed a typical anti-MAG reactivity pattern in the immunohistochemistry assays. Immunostaining reactivity was indistinguishable from patients with monoclonal gammopathy associated anti-MAG neuropathy and different from patients with anti-sulfatide antibody- associated neuropathy (Fig. 1C). The intensity of the myelin staining increased significantly after IgM monoclonal gammopathy detection in patients 1 and 2.

## Discussion

In this study, we identified anti-MAG antibodies in four patients fulfilling CIDP diagnostic criteria and no evidence of monoclonal gammopathy. Only one patient had slightly increased total IgM levels at diagnosis, and in two patients we detected an IgM MGUS after 3 and 4 years of follow-up. All patients presented with clinical, electrophysiological and serological features indistinguishable from those described in patients with anti-MAG + MGUSP^3^.

Since their description, anti-MAG antibodies have always been associated to IgM monoclonal gammopathy^4^. Anti-MAG have been shown to be specific for the diagnosis of MGUSP, while they are negative in healthy controls^2^. Indeed, clinical guidelines^5,9^ only recommend to test anti-MAG antibodies in patients with IgM monoclonal gammopathy. Although the association of IgM monoclonal gammopathy and anti-MAG antibodies is very strong, these recommendations likely generate a selection bias. Our observations suggest that there is a subset of patients with anti-MAG + polyneuropathy without any detectable monoclonal gammopathy that may remain undiagnosed. A few other cases of anti-MAG neuropathy in the absence of monoclonal gammopathy have been described^6–8^, supporting our observations.

In two of our patients an IgM monoclonal gammopathy was detected by serum immunofixation years after diagnosis, and in patient 1 it clearly coincided with an increase in anti-MAG titers. Longer follow-ups may lead to detectable gammopathy in the other two patients but this remains to be confirmed. This phenomenon may imply that, either early IgM gammopathy is not detectable with current immunofixation techniques or that an early antigen-driven autoimmune process is subsequently followed by a clonal expansion and appearance of the monoclonal gammopathy. Whatever the case, these two patients suggest that anti-MAG antibody polyneuropathy displays a spectrum of disease that includes patients that test negative for the presence of gammopathy. Either early testing of anti-MAG or repeated testing of monoclonal gammopathy by immunofixation have to be considered then, especially in patients with clinical and electrophysiological features resembling typical anti-MAG+ MGUSP.

The prevalence of anti-MAG+ patients in our CIDP cohort (5.8%) was similar to that recently reported by a Japanese group (5.7%)^8^. It is also comparable to the amount of anti-NF155+ patients in our study population (4/69) and the prevalence of anti-NF155+ patients reported in other CIDP cohorts^10^. These findings support the concept that CIDP is a heterogeneous disease in terms of immunopathology, clinical presentation and treatment response. Therefore, autoantibody profiling, including detection of anti-MAG antibodies, is useful to guide diagnosis, prognosis and treatment selection in patients with chronic demyelinating neuropathy.

The treatment strategy in anti-MAG associated neuropathies is limited due to the low response rate to current therapies. Treatment with IVIg, plasma exchange, prednisone or rituximab have shown benefits in some patients^3,11^. Two of our patients were initially diagnosed of seronegative CIDP and unsuccessfully treated with immunosuppressant drugs such as azathioprine and cyclosporine that are not considered effective in anti-MAG+ MGUSP. Thus, within the standard therapies used in CIDP, anti-MAG antibodies helped us choose those therapies that could yield better results (e.g IVIg). It would be interesting to assess in larger cohorts if early treatment of these patients with B-cell depleting therapies, such as rituximab, would be more efficacious than if patients are treated after the development of the monoclonal gammopathy^11,12^. Moreover, due to the association of anti-MAG antibodies to the presence of MGUS, all four patients underwent hematological follow-up to study the appearance of monoclonal gammopathy.

In conclusion, we report four patients with anti-MAG neuropathy in the absence of IgM-monoclonal gammopathy. Given these observations, we suggest to test anti-MAG antibodies in patients with chronic demyelinating neuropathy, regardless of the detection of IgM monoclonal gammopathy, especially in those with distal, sensory-ataxic involvement.

## Methods

### Patients, informed consent and protocol approvals

Patients prospectively observed during routine neuromuscular practice between 2007–2017 fulfilling EFNS/PNS diagnostic criteria for CIDP were included. We tested the presence of anti-MAG antibodies in serum. Patients with antibodies towards neurofascin-155 (NF155), nodal neurofascin-140 and 186 (NF140/186), contactin-1 (CNTN1), contactin-1/caspr-1 complex (CNTN1/CASPR1) were excluded from the seronegative cohort. This study was conducted according to a protocol approved by the Institutional Ethics’ Committee of the Hospital de la Santa Creu i Sant Pau. All experiments were performed in accordance with the relevant guidelines and regulations. Written informed consent were obtained from all subjects.

### Clinical and neurophysiological features

In anti-MAG+ patients we collected the age at onset, sex, past medical history and clinical manifestations including initial symptoms and the presence of limb weakness (proximal/distal), gait ataxia or intention tremor. We analyzed neurophysiological findings including motor distal latencies, nerve conduction velocities, F-wave latencies, and the presence of conduction blocks, temporal dispersion, reduced CMAPs or reduced SNAPs. We also collected therapies and response to them.

### Antibody assays

The presence of monoclonal gammopathy (IgA, IgG or IgM) was evaluated by serum protein electrophoresis and serum immunofixation electrophoresis studies (Sebia, France) at diagnosis and follow-up. Antibodies against NF155, NF140/186, CNTN1 and CNTN1/CASPR1 were investigated by immunocytochemistry as previously described^13^. Anti-MAG antibodies were tested by ELISA (Bühlmann laboratories AG, Schönenbuch, Switzerland). We used a cut-off value of 1000 Bühlmann Titer Units (BTU), according to the manufacturer’s instructions. In anti-MAG+ patients, antibodies to sulfatides and gangliosides were also investigated by ELISA as previously described^14^. Further, total levels of IgM in serum were investigated (Immage 800 Nephelometer Beckman Coulter).

### Immunohistochemistry

Monkey peripheral nerve tissue slides (Inova Diagnostics, Inc., San Diego, CA) were blocked with 5% normal goat serum in PBS, incubated with patients’ sera at 1:10 for 1 hour at room temperature, washed and incubated with Alexa Fluor 594 goat antihuman IgM secondary antibody at 1:1000 for 1 hour. Slides were mounted with Fluoromount medium (Sigma-Aldrich, St. Louis, MO). Immunostaining patterns were analyzed and compared with controls. Sera from patients with anti-MAG+ MGUSP and anti-MAG- sulfatides + MGUSP were used as disease controls.

## Supplementary information

Supplementary Table 1.

## Data Availability

All data generated or analyzed during this study are included in this published article (and its Supplementary Information Files).
