# Author Correction: Clinical and laboratory features of anti-MAG neuropathy without monoclonal gammopathy

**DOI:** 10.1038/s41598-021-81911-3

**Published:** 2021-02-10

**Authors:** Elba Pascual-Goñi, Lorena Martín-Aguilar, Cinta Lleixà, Laura Martínez-Martínez, Manuel J. Simón-Talero, Jordi Díaz-Manera, Elena Cortés-Vicente, Ricard Rojas-García, Esther Moga, Cándido Juárez, Isabel Illa, Luis Querol

**Affiliations:** 1 Neuromuscular Diseases Unit, Department of Neurology, Hospital de la Santa Creu i Sant Pau, Universitat Autònoma de Barcelona, Barcelona, Spain; 2 Centro para la Investigación Biomédica en Red en Enfermedades Raras (CIBERER), Madrid, Spain; 3 Department of Neurology, Hospital de la Santa Creu i Sant Pau, Universitat Autònoma de Barcelona, Barcelona, Spain; 4 Department of Immunology, Hospital de la Santa Creu i Sant Pau, Universitat Autònoma de Barcelona, Barcelona, Spain

Correction to: *Scientific Reports* https://doi.org/10.1038/s41598-019-42545-8, published online 16 April 2019

The Acknowledgements section in this Article is incomplete.

“This project was supported by Fondo de Investigaciones Sanitarias (FIS), Instituto de Salud Carlos III, Spain and FEDER under grant FIS16/00627, and personal grant SLT006/17/00131 of the Pla estratègic de recerca i innovació en salut (PERIS), Departament de Salut, Generalitat de Catalunya, IP Luis Querol.”

should read:

“This project was supported by Fondo de Investigaciones Sanitarias (FIS), Instituto de Salud Carlos III, Spain and FEDER under grant FIS16/00627, and personal grant SLT006/17/00131 of the Pla estratègic de recerca i innovació en salut (PERIS), Departament de Salut, Generalitat de Catalunya, IP Luis Querol. The Authors would like to thank the Department of Medicine, Universitat Autònoma de Barcelona, Spain for the support in the development of this work.”

