# Supplementary material for: Clinical and laboratory features of anti-MAG neuropathy without monoclonal gammopathy
Source: Sci Rep. 2019 Apr 16;9:6155. doi: 10.1038/s41598-019-42545-8 (PMC6468000; doi:10.1038/s41598-019-42545-8)
Supplement: Supplementary file 1 — Supplementary Table 1. [file 41598_2019_42545_MOESM1_ESM.pdf]

SUPPLEMENTARY FILE

Title: **Clinical and laboratory features of anti-MAG neuropathy without monoclonal gammopathy**

Elba Pascual-Goñi MD[1], Lorena Martín-Aguilar MD[1], Cinta Lleixa[1], Laura Martínez-Martínez PhD[4], Manuel J. Simón-Talero[3], Jordi Diaz-Manera MD PhD[1,2], Elena Cortés-Vicente MD PhD[1,2], Ricard Rojas-Garcia MD PhD[1,2], Esther Moga MD PhD[4], Cándido Juárez MD PhD[4], Isabel Illa MD, PhD[1, 2], Luis Querol MD, PhD[1, 2] *

Additional file 1: Nerve conduction studies

a) Patient 1

Motor conduction studies

	**Distal Motor Latency, ms**		**Amplitude, mV**		**Conduction Velocity, m/s**	
Nerve	Values	Normal values	Values	Normal values	Values	Normal values
Median (wrist), L	6.6	<3.9	10.7	>6	47	>50
Ulnar (wrist), L	5.8	<3.3	6.5	>5	52	>48
Peroneal (ankle), L	20.3	<5	0.4	>2	MD	>42

Sensory conduction studies

	**Amplitude, µV**		**Conduction velocity, m/s**	
Nerve	Values	Normal values	Values	Normal values
Median (palm), L	33	>25	50	>47
Ulnar (wrist), L	23	>18	40	>45
Sural (lower leg), L	10	>9	33	>40

F-waves studies

	**F Latency, ms**	
Nerve	Values	Normal values
Median, L	36.1	< 30
Ulnar, L	38.7	< 31

b) Patient 2

Motor conduction studies

	Distal Motor Latency, ms		Amplitude, mV		Conduction Velocity, m/s	
Nerve	Values	Normal values	Values	Normal values	Values	Normal values
Median (wrist), R	5.9	<3.9	5.1	>6	44	>50
Tibial (ankle), R/L	10.8/9.5	<6	0.2/0.3	>3	19/22	>38
Peroneal (ankle), R/L	9.1/7.9	<5	1.1/1.4	>2	23/28	>42

Sensory conduction studies

	Amplitude, µV		Conduction velocity, m/s	
Nerve	Values	Normal values	Values	Normal values
Radial (forearm), R	6	>11	50	>47
Sural (lower leg), L	3	>5	39	>38

F-waves studies

	F Latency, ms	
Nerve	Values	Normal values
Median, R	46.4	< 30
Ulnar, R	45.8	< 31

c) Patient 3

Motor conduction studies

	**Distal Motor Latency, ms**		**Amplitude, mV**		**Conduction Velocity, m/s**	
Nerve	Values	Normal values	Values	Normal values	Values	Normal values
Median (wrist), R	4.6	<3.9	5.7	>6	51	>50
Ulnar (wrist), R	3.4	<3.3	8.5	>5	59	>48
Peroneal (ankle), R	4.7	<5	2.2	>2	44	>42
Tibial (ankle), R	3.7	<6	8.4	>3	42	>38

Sensory conduction studies

	**Amplitude, µV**		**Conduction velocity, m/s**	
Nerve	Values	Normal values	Values	Normal values
Ulnar (wrist), R	11.7	>15	39	>42
S Peroneal (lat leg), R	2.7	>6	38	>39

F-waves studies

	**F Latency, ms**	
Nerve	Values	Normal values
Ulnar, R	32.6	< 27
D peroneal, R	58.4	< 47
Tibial, R	59.9	<48

d) Patient 4

Motor conduction studies

	Distal Motor Latency, ms		Amplitude, mV		Conduction Velocity, m/s	
Nerve	Values	Normal values	Values	Normal values	Values	Normal values
Median (wrist), R	10	<3.9	5.4	>6	19	>50
Ulnar (wrist), R	9.2	<3.3	2.6	>5	18	>48
Peroneal (ankle), R/L	NA/NA	<5	NR/NR	>2	NA/NA	>42

Sensory conduction studies

	Amplitude, µV		Conduction velocity, m/s	
Nerve	Values	Normal values	Values	Normal values
Median (wrist), R	NR	>16	NA	>44
Ulnar (wrist), R	NR	>15	NA	>42
Radial (forearm), R	NR	>11	NA	>47
Sural (lower leg), L	NR	>5	NA	>38

F-waves studies

	F Latency, ms	
Nerve	Values	Normal values
Ulnar, R	NR	< 31

MD, missing data; NA not applicable; NR, No response.
